# Improper Use of Antipyrin, Etc.

**Published:** 1891-04

**Authors:** S. Leard Keown

**Affiliations:** Comanche, Texas


For Daniel's Texas Medical Journal.

### IMPROPER USE OF ANTIPYRINE, ANTIFEBRINE, PHENACETINE AND ACETANILID.

BY S. LEARD KEOWN, M. D., COMANCHE, TEXAS.

HIGH FEBRILE ACTION being so common a symptiom in most diseases engaging our attention, it is not surprising that these agents, capable as they are of reducing an elevated temperature so rapidly, and therefore used so much, are occasionally misapplied. When these remedies made their first appearance, a few years ago, I took to their use with some degree of caution, believing that any agent causing such rapid decline of temperature inevitably made a considerable impression in some way upon the system. The rule I at first adopted, and have ever since observed, as to their use, is to restrict their employment to cases of high febrile action having a hot, dry skin and full bounding pulse, always taking the temperature before giving a dose.

The physiological action of these coal tar products is very nearly the same. Having confined myself to the use of acetanilid almost to the exclusion of the others, which I have done because of its cheapness, and believing it fully as good, I am not prepared to discuss the points of difference said to exist between them. Taken into the stomach, they are quickly absorbed by the blood, where their primary action may be said to occur. Here they break down the corpuscular elements, arresting their movements, so that whatever abnormal element the blood is at the time charged with, the same is cut off from the bodily tissues, and the process of repair arrested, temporarily suspending the heat-making process. Their secondary action is upon the nerve centers of the cerebro-spinal and sympathetic systems, which are depressed, allowing of vascular expansion which spreads the blood over a larger area, allowing it to cool more rapidly. Coupling together the primary and secondary actions of the drugs in question, both of which have a decided tendency to lower bodily heat, we can easily understand why they are so energetic in producing their results.

The constant and free use of these remedies in a case of continued fever is calculated to very materially damage the blood, should the nerve centers happily escape their secondary action. In any condition of fever where there is cold surface and slight relaxation of the vaso-motor centers, evinced by low arterial tension, it is dangerous to use either of the articles in question, notwithstanding you may have a considerable amount of fever to combat. A few words may be said here of their use in headaches, where they have proven themselves of great value. Where headache is due to a determination of blood to the brain, to contraction of the capillary system giving rise to passive hyperæmia or to some abnormal material in the blood, these agents are indeed of very great value. There is a class of headaches very often met with in overworked persons in which we find a want of tone, and relaxation of the nerve centers; here we should be careful not to use them.

This subject may be more clearly elucidated by reporting a few cases coming under my observation:

CASE I.—A little girl, age six, had remittent fever. Not until the sixth day was a physician called, who found her temperature quite high, for which he left several doses of antifebrine, with directions to give a powder every two hours till fever was reduced. The first dose caused free perspiration, and no doubt would have benefited the patient if it had been followed by quinine. The parents not having used the drug before, and being poor nurses, not knowing, really, whether the fever was declining or not, gave the second dose very soon, after which the symptoms became alarming. The attending physician being away at this time, I was called, and found the patient in a profound state of collapse from which she never rallied, although bimuriate of quinine was used hypodermically, alcoholic stimulants freely used, bottles filled with warm water put near the body and mustard plasters applied to the surface of the spine and extremities. As soon as the attending physician returned, we used strichnia hypodermically. By these means she was kept alive twenty hours, finally dying in a collapsed state.

CASE II.—A young man about nineteen, had malarial continued fever which lasted about four weeks. He was attended by a physician of more than average ability, the treatment used being proper in every detail, except there was a too free use of antipyrine. When the fever subsided he was anæmic, and notwithstanding good feeding and appropriate nursing, he died from exhaustion, after having been clear of fever for about ten days.

CASE III.—Little boy about four years old had catarrhal continued fever. He had good medical attention and careful nursing. A free use of acetanilid was made, till on one occasion considerable depression followed after a dose was given, after which it was used with more caution. For seveyal days before he died the temperature would advance after a dose of acetanilid was given. His fever suddenly rose about twenty-four hours before his death. The drug was again used, which was followed by an anaemic state of the brain with fever advancing to 106° in which condition he died.

CASE IV.—A lady about forty was taken with remittent fever.

On the fourth day the temperature, which had previously ranged from 103° to 105°, went to 107°. Acetanilid had been freely used, and at this rise of temperature the remedy was *increased.* Two hours after the temperature had advanced to 107° it reached 109°. The wet pack—ice locally applied—and every available means was used to reduce the temperature, which fell to 106°, where it remained till death, which occurred in a few hours.

CASE V.—Boy, age 11 had malarial continued fever. Was attended by a skillful physician, and had splendid nursing. About the fifteenth day his fever ran higher. Acetanilid was given by his parents; collapse followed, from which he never recovered, though he lived over twenty-four hours.

CASE VI.—A young lady about twenty years of age, suffering with malarial continued fever. Her symptoms were of mild character during the first three weeks, fever ranging from 101° to 102°. During the fourth week fever advanced to 103°. The attending physician had not seen her in several days, and her parents, attempting to réduce the fever, gave a dose of antipyrine, soon after which her temperature rose to 106°. The doctor was called, who found, upon reaching her, a cyanosed condition of the skin, weak, irregular pulse, cold extremities and chilliness of the surface. At his suggestion consultation was called for, and I reached the case a few hours later, by which time her condition had improved uuder the use of bottles filled with warm water placed around her body, and friction applied to the surface. As the circulation continued to improve the fever declined to 102°. A few hours later, fever rose to 105°, for which a moderate dose of antipyrine was given, which again produced slight cyanosis without reducing the fever. No further use of the drug was made in her case, baths being resorted to for advancing temperature, and she is, at time of this writing, fairly convalescing.

I do not wish to create a prejudice against these potent remedies, which I consider a valuable addition to our materia medica; but only desire to caution those using them against carelessness in their application. The condition of the patient, as already referred to, must be observed, and the effects of the drug carefully noticed. The practice which has grown so common among laity of using these agents, indiscriminately, should be denounced as dangerous, When used by a competent physician, capable of observing the conditions of the patient, especially as relates to the circulation, and one who understands their physiological action, no harm may be expected to follow their use, but instead we can expect to accomplish a great deal by way of reducing fever and relieving pain.

